# Editorial

**Published:** 1869-04

**Authors:** 


### Editorial.

---

STATE MEDICAL SOCIETY.—Let all our readers remember that the next Annual Meeting of the Illinois State Medical Society is to be held in Chicago on the *third* Tuesday in May. All Societies are entitled to one delegate for every five members.

---

AMERICAN MEDICAL ASSOCIATION.—Arrangements have been completed by which all Delegates to the meeting in New Orleans on the first Tuesday in May next, can be returned *free* over either of the railroad routes from New Orleans to Chicago and intermediate places. One route goes by way of Louisville and Nashville, and will afford an opportunity to visit the Mammoth Cave in Kentucky; and the other is by way of Cairo, Columbus, etc.

The fare over both routes from Chicago to New Orleans is the same, $38.00, and must be paid in going, the Delegate being allowed to pass back over the same route free. These are very liberal arrangements on the part of the railroads, and should ensure a large delegation from the North-west.

---

ALUMNI ASSOCIATION OF CHICAGO MEDICAL COLLEGE.—This Association held its second anniversary meeting in the College on the evening of the 22d of March. It was numerously attended, and the full proceedings will be published in our next number.

---

Death of Dr. A. A. Dunn.—The undersigned committee, to whom was referred the duty of expressing the sentiments of this Society regarding the late A. A. Dunn, M.D., of this city, would respectfully report:—

That Dr. Dunn, by his natural intellectual endowments, his education, his professional skill and integrity, his ardent support of Medical Societies and ethics, had won the esteem and confidence of all the members of the profession who knew him; while the addition of a uniform Christian deportment, and a self-sacrificing patriotism, had added equally the respect and warm attachment of all classes of community.

That, by his death, the Profession and Society have lost one of their most respected and useful members, the nation one of its true *heroes* and defenders, and this Society one we all esteemed as a brother.

*Resolved*, That a copy of the foregoing expression of sentiment be communicated to the family of the deceased and to the medical journals and daily papers of this city.

N. S. Davis,

T. D. Fitch, } *Committee.*

A. Groesbeck,

---

Death of Dr. John Charlton.—At a meeting of the Physicians of Freeport, held March 16, 1869, the following preamble and resolutions were unanimously adopted:—

Whereas, It has pleased Almighty God to remove from among us Dr. John Charlton, an old and honored member of our profession.

*Resolved*, That by the death of Dr. Charlton the profession has lost a member whose skill and experience entitled him to our regard and respect, whose uniform kindness and courtesy challenged our affection, and whose honesty and integrity as a Physician gave him a deservedly high rank in his profession.

*Resolved*, That in the death of Dr. CHURLTON, society has lost a useful citizen, and his family a kind and affectionate husband and father.

*Resolved*, That to the family of the deceased we tender our heartfelt sympathy.

F. W. HANCE,

W. J. MCKIM,

L. A. MEUSE, } *Committee.*

---

### COMMENCEMENT EXERCISES IN CHICAGO MEDICAL COLLEGE.

The Tenth Annual Commencement in the Chicago Medical College was held in the College Hall on Monday and Tuesday, March 22d and 23d, 1869. The exercises on Monday commenced at 3 o'clock, and occupied the remainder of the afternoon. They consisted in the reading of Inaugural Thesis by HENRY H. SLOAN, GEORGE K. DYAS, and WILLIAM E. QUINE; and a public examination of the candidates for graduation in class.

The exercises on Tuesday commenced at 3 o'clock P.M.; and we clip the following brief account from the daily *Chicago Times*:—

The commencement exercises of the tenth graduating class of the Chicago Medical College took place in the public hall of the College, No. 1015 State Street, at 3 o'clock on yesterday afternoon. The hall was densely filled with the friends of the institution.

The President, Dr. N. S. Davis, occupied the Chair.

Prayer was offered by the Rev. Charles E. Cheney.

All the undergraduates who were present, in accordance with the custom of the College, came forward and received their certificates for industry and scholarship. Dr. N. S. Davis addressed them a few words, remarking that they should remember that they had but just entered upon the wide domain of medical knowledge, and could only obtain the reward of final success by untiring industry and perseverance.

The Professor of Surgery, Dr. E. Andrews, next made a distribution of hospital certificates to all those entitled to receive them.

The President then awarded the prizes adjudged by a committee to the writer of the best and second best thesis on medical subjects. Thomas G. Williams, of Wisconsin, was decided to have written the best thesis; and he received the first premium—"Atkins on the Practice of Medicine." His subject was "The Physiological Action of the Bromides."

The second prize was awarded to Charles N. Cooper, of Illinois, for the second best thesis. His subject was the origin and Uses of Fibrin." He received a work on diseases of children.

The President then conferred the degrees upon the graduating class. Their names are as follows:—

*Ordinary Degrees*—Samuel Alexander, Daniel J. Allaben, Charles Ashworth, William A. Barstow, Geo. W. Barton, Carl Oscar Bendeke, Wallace Blanchard, Dwight E. Burlingame, William H. Carithers, William C. Chafee, Charles N. Cooper, William Deal, Simeon H. Drake, George K. Dyas, J. W. Folke, George H. Fuller, James S. Gibson, George W. Goodner, Chas. S. Hamilton, Green B. Hoblit, Theodore H. Johnson, Edward R. Kittoe, Joseph Kitchen, David T. Martyn, A. B. McCandless, D. Irwin McMillan, Joseph Milliron, Pacificus B. Porter, William E. Quine, Nelson Rinedollar, Isaac P. Sinclair, Henry H. Sloan, Joseph Sterrett, Daniel C. Stillians, Thomas G. Williams.

*Ad Eundem Degrees*—J. H. Curtis, Ernest Stachr.

*Honorary Degrees*—Jacob Holke, J. H. Newland, Meniard Risch, Shubael M. Reynolds.

The President addressed a few remarks to the class, urging them in all their future lives to remember they were acting in a field that concerns health and happiness; and that there were no persons who had opportunities for rendering so much happiness to their fellow men, as the faithful physician. They should remember that there were three things that were absolutely necessary to success: these were industry, integrity, and perseverence.

A very appropriate response on behalf of the class, was delivered by William H. Carithers.

Dr. J. S. Jewell next delivered the valedictory address. The world was divided into two great classes, the laborers of the mine, and the laborers of the mint. In all inductive sciences, there must be two epochs; first, the empirical, and secondly, the constructive and logical period. It is pre-eminently so in medical science. Many succeed in gathering facts, in observing phenomena; but they fail in constructive and logical power. Of the two provinces, the most lamentable failures are in the latter domain. In the study of botany, geology, astronomy, and their kindred sciences, we must suspend for some time our deductions from the phenomena observed; but not so in the science of physiology. It requires, therefore, a power of quick, logical deduction to be a good physician; and this can only be attained by long and laborious reflection upon the facts already discovered. The process of logic to be relied upon is not that of the syllogism; but rather that of Bacon and Whewell, the logic of the inductive sciences. A knowledge of languages will not provide this; nor will a ready memory. The Professor proceeded to show what might be considered a true method of logic. The address was listened to with deep interest.

After the benediction, the assembly dispersed.

The programme was completed with a very pleasant social entertainment in the evening, at the residence of Prof. N. S. Davis.

---

East India Opium.—At Patna is one of the two great opium factories of India. It is the greater of the two, and may, therefore, be safely styled the largest poisoning agency in the world. The establishment faces the river Ganges, whose bed is here four miles across—at this season a desert of caked mud, with the river far away on the other side of the waste. The opium is shipped to Calcutta in a steamer, and it is a good instance of the fickleness of Indian rivers—those plagues of engineers—that last year, and for many years before, the sacred stream ran so close to Patna, that wharves were erected from which the chests could be put right on the steamers, and where the timber, wherewith to make the next year's chests, could be landed. This year the chests have to be carried a mile or so before being shipped.

This opium-packing for 1867 was just over at Christmas, and nearly 30,000 chests of China opium had been sent down to Calcutta, worth about £4, 000,000. Each chest contains 40 cakes —the dark, sticky stuff, ingeniously inclosed in a coating of dried poppy-leaves, so that each cake (weighing about two pounds) presents the appearance of a Dutch cheese or a cannon-ball.

It has given rise to the saying, that in war the British gave the Chinese cannon-balls of iron, and in peace cannon-balls of opium, thus giving them the choice of being shot or poisoned, and making them pay sharply for either attention. In return for this, they feed us with tea and clothe us in silk, which seems to show a truly celestial spirit.—*Sci. Amer.*—*Med. and Surg. Reporter.*

### Mortality for the Month of February, 1869:—

### CAUSES OF DEATH.

| | | | | | |
|---|---|---|---|---|---|
| Accidents, burns | 1 | Exposure | 3 | Meningitis, and brain, | |
| " drowned | 1 | Erysipelas | 4 | dropsy of | 2 |
| " poisoned | 1 | Fever, congestive | 1 | " cerebro-spi- | |
| " R. R. | 2 | " puerperal | 6 | nal | 3 |
| Albuminuria and | | " scarlet | 37 | Metritis | 2 |
| dropsy | 1 | " " and diph- | | Old age | 7 |
| Anæmia | 1 | theria | 4 | Paralysis | 2 |
| Apthæ | 2 | " " erysipelas | 1 | " lungs, hemor- | |
| Apoplexy | 6 | " " nephritis | 1 | rhage of | 1 |
| " and intem- | | " " malignant | 1 | Peritonitis | 1 |
| perance | 1 | " typhoid | 6 | Phthisis pulmonalis | 33 |
| Asthma | 1 | " " heart dis- | | " " old age | 1 |
| Births, premature | 14 | ease | 1 | Pneumonia | 31 |
| " still | 44 | Gastritis | 1 | " and dropsy | 1 |
| Bowels, cancer of | 1 | " and dropsy | 1 | " whooping- | |
| Brain, congestion of | 4 | Glands, parotid, inflam- | | cough | 1 |
| " disease of | 1 | mation of | 1 | Pneumonia broncho | 3 |
| " inflammation of | 2 | Heart, disease of | 3 | " pluero | 1 |
| " softening of | 1 | " and kidneys, | | " typhoid | 5 |
| Bronchitis | 5 | fatty degenera- | | Scrofula | 2 |
| " capillary | 1 | tion of | 1 | Small-pox | 1 |
| Cellular tissues, indur- | | " valvular disease | 1 | Sub-maxillary glands, | |
| ation of | 1 | Hepatitis and apoplexy | 1 | abscess of | 1 |
| Cholera infantum | 1 | Hernia, scrotal, stran- | | Spine, irritation of | 1 |
| Cordis, hypertrophy | 1 | gulated | 1 | Stomach, cancer of | 1 |
| Convulsions | 32 | Hydrothorax | 2 | Suicide by laudanum | 1 |
| " sore mouth | 1 | Hydrocephalus | 2 | " " shooting | 1 |
| Croup | 4 | " acute | 1 | Talus mesenterica | 5 |
| " membranous | 1 | Inanition | 3 | " universalis | 1 |
| Cyanosis | 1 | Injuries by fall | 1 | Teething | 1 |
| Debility | 1 | Indigestion | 1 | " convulsions | 1 |
| Diarrhœa | 3 | Icterus infantum | 1 | Unknown, | 4 |
| " chronic | 3 | Intemperance and ex- | | Uterus, cancer of | 3 |
| Diphtheria | 8 | posure | 1 | Varioloid | 1 |
| " brain, con- | | Kidneys, Bright's dis- | | Vertebare, caries of | 1 |
| gestion of | 1 | ease of | 3 | Varicella and convul- | |
| " bronchitis | 1 | " inflammation | 1 | sions | 1 |
| Dropsy | 3 | Liver, cancer of | 1 | Whooping-cough | 8 |
| Dyspepsia | 1 | " disease of | 1 | " convulsions | 1 |
| Dysentry | 2 | " inflammation of | 1 | " pneumonia | 1 |
| " and brain, | | Lungs, congestion of | 3 | Wounds of throat, self- | |
| congestion of | 1 | Measles | 1 | inflicted | 1 |
| Encephalitis | 1 | Meningitis | 4 | | |
| Enteritis | 3 | | | Total | 333 |

### AGES.

| | | | | | |
|---|---|---|---|---|---|
| Under 1 | 85 | 20 to 30 | 30 | 70 to 80 | 12 |
| 1 to 3 | 63 | 30 to 40 | 34 | 80 to 90 | 3 |
| 3 to 5 | 30 | 40 to 50 | 20 | Unknown | 1 |
| 5 to 10 | 18 | 50 to 60 | 10 | | |
| 10 to 20 | 6 | 60 to 70 | 21 | Total, | 333 |

### COMPARISON.

Deaths in Feb., 1869, 333 | Deaths in Feb., 1868, 425 | Decrease, 92

Deaths in Jan., 1869, 382 | Decrease, 49

| | | | | | |
|---|---|---|---|---|---|
| Males, | 164 | Females, | 169 | Total, | 333 |
| Single, | 231 | Married | 102 | Total, | 333 |
| White, | 327 | Colored, | 6 | Total, | 333 |

NATIVITIES.

| | | | | | |
|---|---|---|---|---|---|
| Bohemia, | 2 | England, | 10 | Sweden, | 5 |
| Canada, | 5 | France, | 1 | Scotland, | 4 |
| Native Chicago, | 56 | Germany, | 46 | Unknown, | 2 |
| Foreign " | 103 | Ireland, | 34 | | |
| U. S., other parts, | 59 | Norway, | 6 | Total, | 333 |

MORTALITY BY WARDS FOR THE MONTH.

| Ward. | Mortality. | Pop. in 1868. | One death in | Ward. | Mortality. | Pop. in 1868. | One death in |
|---|---|---|---|---|---|---|---|
| 1 | 4 | 9,094 | 2,273½ | 14 | 20 | 14,839 | 742 |
| 2 | 17 | 13,074 | 769 | 15 | 22 | 21,078 | 958 1-10 |
| 3 | 19 | 15,076 | 793½ | 16 | 21 | 15,465 | 736 3-7 |
| 4 | 19 | 17,796 | 936[illegible] | County hosp. | 7 | | |
| 5 | 17 | 16,033 | 943⅛ | Accidents, | 5 | | |
| 6 | 17 | 13,083 | 769 1-10 | Mercy Hosp. | 1 | | |
| 7 | 33 | 25,492 | 772½ | Suicides, | 2 | | |
| 8 | 20 | 15,813 | 790 4-7 | Immigrants | 3 | | |
| 9 | 28 | 19,297 | 689⅙ | Soldier's Ho. | 1 | | |
| 10 | 9 | 12,925 | 1,435 7-9 | Prot. Orph. Asylum | 1 | | |
| 11 | 14 | 14,340 | 1,024 1-5 | Home for the Friendless, | 2 | | |
| 12 | 29 | 17,485 | 603 | | | | |
| 13 | 22 | 11,164 | 507½ | | | | |

Total, ---------------------------------------- 374

The decrease in the mortality of this month, compared with the corresponding month of 1868, is quite remarkable, indicating a good condition of health. This is most marked in the diminution of deaths by acute inflammatory diseases, particularly those of the lungs, infantile diseases, old age, and small-pox. In only two diseases has there been an increase, *viz.*, scarlet fever and erysipelas. This diminution in the mortality is owing to the remarkably mild and equable temperature of the month, and our comparative exemption from contagious diseases.

---

Money Receipts to March 17th.—Drs. A. D. Fitch, $6; F. Merriman, 9; Jacob Shnech, 3; D. Newcomb, 10; T. A. Lilly, 3; C. T. Hunter, 3; J. F. Young, 3; E. R. Atwood, 3; B. Lee, 3; F. R. Payne, 3; H. C. Lester, 3; D. Scott, 3; W. H. Buchtel, 3; Hiram Nance, 3; Brown & Kimber, 6; M. T. Didlake, 3; Thos. Bevan, 3; N. Lenn, 4 50; A. B. McCandless, 3; O. T. Maxon, 3; D. H. Patton, 6; L. M. Triplett, 3; A. G. Greenman, 3; Chas. Hill, 5; L. D. Smedley, 3; I. V. Golton, 3; S. P. Breed, 3; G. C. Paoli, 4; Wm. H. Cook, 3; L. D. Tompkins, 3; P. A. Shotwell, 3; D. B. Trimble, 3; Thos. Winston, 5; M. T. Didlake, 3; C. S. Ford, 5; Geo. F. Hunt, 3; H. S. Hall, 6 50; H. W. Boyd, 6; J. D. Mitchell, 5; Herbert Harris, 6; D. C. Stillions, 3; — Risch, 3; Thos. W. Howes, 3; John Bell, 3; Wm. Martin, 1; J. Priestman, 3; —Newland, 3; J. F. Williams, 9; Wm. Horne, 6; Saml. Alexander, 1 50; J. F. Kelsey, 3.

### AMERICAN MEDICAL ASSOCIATION.

OFFICE OF PERMANENT SECRETARY:

WM. B. ATKINSON, M.D., S. W. cor. Broad and Pine Streets, Philadelphia.

The Twentieth Annual Session will be held in NEW ORLEANS, La., May 4th, 1869, at 11 A.M.

The following Committees are expected to report:—

On Diseases of the Cornea.—Dr. Jos. S. Hildreth, Illinois, Chairman.

On Cultivation of the Cinchona Tree.—Dr. Lemuel J. Deal, Pennsylvania, Chairman.

On Excision of Joints for Injuries.—Dr. J. B. Reid, Georgia, Chairman.

On Alcohol and its Relations to Medicine.—Dr. John Bell, Pennsylvania, Chairman.

On the Cryptogamic Origin of Disease, with special reference to recent microscopic investigations on that subject.—Dr. Edward Curtis, U. S. A., Chairman.

On Operations for Hare-lip.—Dr. A. Hammer, Missouri, Chairman.

On Clinical Thermometry in Diphtheria.—Dr. Jos. G. Richardson, New York, Chairman.

On Prophylactics in Zymotic Diseases.—Dr. Nelson L North, New York, Chairman.

On Inebriate Asylums.—Dr. C. H. Nichols, D. C., Chairman,

On the Influence of the Pneumogastric Nerve on Spasmodic and Rhythmical Movements of the Lungs.—Dr. Thomas Antisell, D. C., Chairman.

To Examine into the Present Plan of Organizatiou and Management of the United States Marine Hospitals.—Dr. D. W. Bliss, D. C., Chairman.

On the Utilization of Sewerage.—Dr. Stephen Smith, New York, Chairman.

On the Influence of Quarantine in Preventing the Introduction of Disease into the Ports of the United States.—Dr. Elisha Harris, N. Y., Chairman.

On Nurse Training Institutions.—Dr. Samuel D. Gross, Pennsylvania, Chairman.

On Commissioners to aid in Trials involving Scientific Testimony.—Dr. John Ordronaux, N. Y., Chairman.

On Annual Medical Register.—Dr. John H. Packard, Pennsylvannia, Chairman.

On Devising a Plan for the Relief of Widows and Orphans of Medical Men.—Dr. John H. Griscom, N. Y., Chairman.

On Veterinary Colleges.—Dr. Thomas Anticell, D. C., Chairman.

On Specialities in Medicine, and the Propriety of Specialists Advertising.—Dr. E. Lloyd Howard, Maryland, Chairman.

On Library of American Medical Works.—Dr. J. M. Toner, D. C., Chairman.

On Vaccination.—Dr. Henry A. Martin, Massachusetts, Chairman.

On the Decomposition of Urea in Uræmic Poisoning.—Dr. H. R. Noel, Maryland, Chairman.

On the best method of Treatment for the different forms of Cleft Palate.—Dr. J. R. Whitehead, N. Y., Chairman.

On Rank of Medical Men in the Navy.—Dr. N. S. Davis, Illinois, Chairman.

On Medical Ethics.—Dr. D. Francis Condie, Pennsylvania, Chairman.

On American Medical Necrology.—Dr. C. C. Cox, Maryland, Chairman.

On Medical Education.—Dr. J. C. Reeve, Ohio, Chairman.

On Medical Literature.—Dr. E. Warren, Maryland, Chairman.

On Prize Essays.—Dr. S. M. Bemiss, Louisiana, Chairman.

On the Climatology of—Maine, Dr. J. C. Weston; New Hampshire, Dr. P. A. Stackpole; Vermont, Dr. Henry Janes; Massachusetts, Dr. H. I. Bowditch; Rhode Island, Dr. C. W. Parsons; Connecticut, Dr. E. K. Hunt; New York, Dr. W. F. Thoms; New Jersey, Dr. Ezra M. Hunt; Pennsylvania, Dr. D. F. Condie; Maryland, Dr. O. S. Mahon; Georgia, Dr. Juriah Harriss; Missouri, Dr. George Engelman; Alabama, Dr. R. F. Michal; Texas, Dr. T. J. Heard; Illinois, Dr. R. C. Hamil; Indiana, Dr. J. F. Hibberd; District of Columbia, Dr. T. Antisell; Iowa, Dr. J. C. Hughes; Michigan, Dr. Abm. Sager; Ohio, Dr. T L. Neal; California, Dr. F. W. Hatch; Tennessee, Dr. B. W. Avent; West Virginia, Dr. E. A. Hildreth; Minnesota, Dr. Samuel Willey; Virginia, Dr. W. O. Owen; Delaware, Dr. L. B. Bush; Arkansas, Dr. G. W. Lawrence; Missisippi, Dr.——Compton; Louisiana, Dr. L. T. Pimm.

Secretaries of all medical organizations are requested to forward lists of their delegates, as soon as elected, to the Permanent Secretary.

Any respectable physician who may desire to attend, but cannot do so as a delegate, may be made a *member by invitation*, upon the recommendation of the Committee of Arrangements.

W. B. ATKINSON.

---

### AMERICAN MEDICAL ASSOCIATION.

MEETING AT NEW ORLEANS, TUESDAY, MAY 4TH, 1869.

I am authorized by the Atlantic and Mississippi Steamship Co., of St. Louis, to say, that they will carry Doctors and their Ladies to attend the Meeting of the Association, at the following rates, viz.:

| | |
|---|---|
| From St. Louis to New Orleans, each Passenger | $20 00 |
| " Cairo " " " " | 18 00 |
| " Memphis " " " " | 15 00 |
| Returning, | |
| From New Orleans to Memphis, each Passenger | $15 00 |
| " " " Cairo, " " | 18 00 |
| " " " St. Louis, " " | 20 00 |

The Company start a first class Steamer from St. Louis every 48 hours, Sundays included, and the usual time from St. Louis to New Orleans, is about six days, and from Cairo to New Orleans, about four and a-half days. Passengers can go on any of their boats at the above rates, which includes meals and staterooms.

The Steamer which will, however, take down the great body of the Doctors, wishing to travel by the river, will leave St. Louis at 5 o'clock P.M., on Wednesday, the 28th of April; Cairo on Thursday evening, after the arrival of the afternoon train on the Illinois Cen. R. R.; and Memphis on Friday evening, reaching New Orleans from Monday noon to Tuesday morning.

Parties arriving by railroad, to take this boat, at either St. Louis, Cairo, or Memphis, had better make their calculations to reach the point of embarkation, at least one train in advance of the time of the boat's departure. But, if any one should arrive at Cairo or Memphis too late for this boat, he will find one or more boats passing for New Orleans every day, at ordinary fare.

It was deemed best to make the arrangement for a definite fare each way, so that one can go either down or up, or both, as he may choose, by the river, and know in advance just what he will have to pay.

To avail himself of this boat, one may apply on board, making it known that he is on his way to attend the Association, or, perhaps better, write me a line as early as convenient, stating how many ladies, if any, will accompany him.

Good Steamers, also leave Louisville for New Orleans every two or three days, occupying from six to seven days in the passage down. If a considerable number of Doctors should wish to take passage from Louisville, and would make application in a body to E. T. Sturgeon, Supt. Louisville & New Orleans Packet Co., at Louisville, or the Captain of a Steamer, starting at the proper time, he would probably give them a liberal reduction from the ordinary fare, which varies from thirty to forty dollars, according to the style and accommodation of the boat.

From Cincinnati no suitable boat can be taken through to New Orleans, but the Cincinnati & Louisville U. S. Mail Line, will take one going to the Association from Cincinnati to Louisville on one of their fine boats, and from thence to New Orleans by rail, for forty dollars, and return him on the same route to Cin. free. Two Mail Boats leave Cincinnati every day at 12 M., and 6 o'clock P.M., except Sundays, one at 12 M. I am not advised as to what arrangements have been made with other railroads.

JAMES F. HIBBERD, M.D., *Richmond, Ind.*

---

SECOND ATTACKS OF SMALL-POX.—It is no new thing for variola to attack persons a second time. Jenner refers to seventeen such cases among the nobility of England, more than half a century ago. Upwards of two hundred such cases were reported in London in 1851. In Wirtemburg 143 cases were recorded, of which 28 died. The proportion after inoculation is still greater. One instance is on record of five children having been inoculated with small-pox, successfully, four of whom subsequently contracted the disease, which was fatal to one of the number. An English surgeon is mentioned as having always an attack of small-pox after attending a patient with the disease.—*Pacific Medical and Surgical Journal.*

---

SIR CHARLES BELL'S DISCOVERIES.—To those interested in the discoveries of Sir Charles Bell, we commend the perusal of his pamphlet—"Idea of a New Anatomy of the Brain"—only three of the original copies of which are known to be extant. Its reprint with letters, now for the first time published, written by the author of the Essay to his brother, George Joseph Bell, between the years 1807 and 1821, in the number of the *Journal of Anatomy and Physiology* for Nov., 1868, is very opportune, as his exact claims have been often misunderstood, and at the present moment are attracting much attention both in this country and abroad.—*Med. News and Library.*

---

PUERPERAL CONVULSIONS.—Dr. Fordyce Barker, in a clinical lecture recently delivered at Bellevue Hospital, summed up our knowledge on this subject as follows:—

First, That we have puerperal convulsions arising from uræmia, caused by Bright's disease of the kidneys.

Second, That we have convulsions associated with congestion of the kidneys and albuminuria, but it is not yet proven that the convulsions and the renal congestion bear the relation of cause and effect.

Third, That we have convulsions that arise from reflex irritation and congestion of the true spinal system, without evidence of any renal affection.

In relation to one point in the treatment, *viz.*, the delivery of the child, the principle which should govern us is this:—Whenever delivery by art can be effected with less irritation than would be produced by the continuance of the child in the parturient canal, it should be resorted to.—*Medical Record*, *Nov. 16*, *1868.*

---

New Mode of Preparing Objects for the Microscope.—M. Rauvier proposes (Archives de Physiologie) a new and simple method, which consists in the employment of picric or carbazotic acid. This acid is only moderately soluble in water, and a saturated solution may therefore be employed. It possesses the further advantage of being very cheap. It is admirably adapted for all tissues containing much blood, and therefore for specimens of liver, lung, etc. It appears to act by effecting coagulation of the albuminous substances, though, unlike alcohol and chromic acid, it does not occasion any fusion of the constituents of the tissue. The red globules retain their form and characters extremely well. The portion of tissues required to be examined should be plunged into the solution, and after the lapse of twenty-four hours it will be found to have acquired sufficient firmness to permit of very fine sections being made with a razor. The saving of time by this method as compared with the chromic acid is immense. The preparations will take color from carminate of ammonia, and may be preserved in glycerine.—*Lancet*, *Nov. 21*, *1868.*—*Med. News and Library.*

---

The *London Lancet* says:—"We know not whether our Scotch friends are more given to indulgence in spirituous liquors than are the English, but they have adopted a sensible and humane method of treating that form of disease—for it amounts to a disease,—an incontrollable love of alcoholic drinks. We have, on former occasions, earnestly called attention to this class of cases, in the interests of the drunkard and his family; but the liberty of the subject has proved a bugbear in the way of all legislation. People are beginning to adopt more healthy notions, however, and the drunkard is not allowed to exercise his liberty at the expense of other people's health and happiness, quite so much as heretofore. We learn that the Lunacy Board of Scotland has adopted some action in the matter. In a report of a former year, an opinion was expressed that persons unable to resist the tendency to excessive drinking should be allowed to place themselves under control and treatment without authority from the sheriff. By the Lunacy Amendment Act, 1866, asylums are authorized to receive for care and treatment any person who expresses in writing to the Commissioners in Lunacy his wish to become a voluntary patient, and obtains their consent; and that this provision was taken advantage of in 1867 in Scotland by 17 persons: fourteen were admitted into public and three into private asylums. We would go a little further, and make it permissible to place such persons under medical restraint, where it could be shown by clear and unmistakable evidence that a drunkard exercised no control over his habits, but was ruining himself in health, and plunging his wife and family into destitution and misery."—*Medical and Surgical Reporter.*

---

Bromide of Potassium for the Sleeplessness of Infants.—M. Moutard-Martin has communicated to the French Academy of Medicine a memoir on "Some Applications of the Bromide of Potassium to the Medicine of Young Infants." Every one, he observes, admits the possession of sedative properties by the bromide, and in this direction it has become one of the most useful substances in the Materia Medica. Bearing in mind its hyposthenic action in erethism of the nervous system, and its innocuity, even in large doses, he believed that it might be employed with advantage in some of the pathological conditions of very young children. Among these, sleeplessness, alike mischievous to the infant and wearying to the nurse, is one of very common occurrence. The child does not seem otherwise ill, but has a very great insufficiency of sleep both by day and night, or only at night. Where a great variety of means has failed to remove this sleeplessness, the bromide succeeds in a remarkable manner, and M. Moutard-Martin adduces in his paper several cases in proof. His conclusions are:—1. The bromide of potassium given in small doses (from five to twenty centigrammes) is very well tolerated by young infants. 2. By its sedative action it cures *insomnia* in these cases. 3. Administered to infants suffering from the accidents of dentition, such as restlessness, *insomnia*, cough, etc., it frequently relieves these; and it is probable that its employment, regulated with prudence, would sometimes prevent the occurrence of convulsions. 4. It should not be administered to infants when suffering from diarrhœa. 5. In certain exceptional cases in which the nervous erethism is predominant, its action is prompt and decisive.—*Medical Times and Gazette, Dec. 12, 1868.—Medical News and Library.*

---

The New Illustrated Edition of Webster's Dictionary.—This seemingly dry and certainly ponderous book has its peculiar charms. Here is collected and tersely set down a vast quantity of various and useful knowledge, such as is indispensable to educated men and women. Here are an hundred and fourteen thousand words, defined with a clearness, fullness, precision, and wealth of illustration, that denote the soundest scholarship, and the most entire fidelity to laborious details. Altogether the work is a marvelous specimen of learning, taste, and thorough labor. We praise it heartily, because we believe it deserves the heartiest praise.—*New York Albion.*

